# Primary Care 2030: Creating an Enabling Ecosystem for Disruptive Primary Care Models to Achieve Universal Health Coverage in Low- and Middle-Income Countries

**DOI:** 10.5334/aogh.2471

**Published:** 2020-02-03

**Authors:** Dan Schwarz, David Duong, Chase Adam, J. K. Awoonor-Williams, Darren Back, Abhay Bang, Rani Bang, Madeleine Beebe, Shreya Bhatt, John Campbell, Magnus Conteh, Dessislava Dimitrova, Donika Dimovska, Jean-Paul Dossou, Timothy Evans, Mazin Gadir, Khaleda Islam, Ronald Kasyaba, Priya Kumar, Catherine Levy, Tran Mai Oanh, Nahed Monsef, Juhwan Oh, Nathaniel Otoo, Daniel Palazuelos, Andy Poh, Sylvana Sinha, Catharine Smith, Ben Stewart, Cicely Thomas, Beth Tritter, Peter Varnum, Taylor Weilnau, Andrew Ellner

**Affiliations:** 1 Harvard Medical School, Program in Global Primary Care and Social Change, Boston, US; 2 Ariadne Labs at Brigham and Women’s Hospital and Harvard T.H. Chan School of Public Health, Boston, US; 3 Watsi, San Francisco, US; 4 Ghana Health Services, Policy, Planning, Monitoring and Evaluation, Accra, GH; 5 Pfizer Inc., Social Investments and Corporate Responsibility, New York, US; 6 Society for Education, Action and Research in Community Health (SEARCH), Gadchiroli, Nagpur, IN; 7 Muso, Bamako, ML; 8 Medic Mobile, Mumbai, IN; 9 Results for Development, Washington, DC, US; 10 Last Mile Health Inc., Community Health Academy, Boston, US; 11 World Economic Forum, Geneva, CH; 12 Centre de recherche en Reproduction Humaine et en Démographie, Public Health, BJ; 13 Instituut voor Tropische Geneeskunde, Public Health, Antwerpen, BE; 14 The World Bank Group, Health, Nutrition and Population Global Practice, Washington, DC, US; 15 Dubai Health Authority, Executive Office for Organizational Transformation, Dubai, AE; 16 Health Services of Bangladesh, BD; 17 Uganda Catholic Medical Bureau, Kampala, UG; 18 Sanofi SA, Global Health Programs for Non Communicable Diseases, Paris, FR; 19 Health Strategy and Policy Institute, Department of Public Health, Hanoi, VN; 20 Dubai Health Authority, Department of Health Affairs, Dubai, AE; 21 Seoul National University College of Medicine, JW LEE Center for Global Medicine, Seoul, KR; 22 Harvard Medical School, Department of Global Health and Social Medicine, Boston, US; 23 Partners In Health, Boston, US; 24 United Arab Emirates Prime Minister’s Office, Dubai, AE; 25 Praava Health, BD; 26 Harvard Medical School, Center for Primary Care, Boston, US; 27 F Hoffmann-La Roche AG, Foundation Medicine, Basel, CH; 28 Primary Health Care Performance Initiative, Washington, DC, US; 29 World Economic Forum, Global Health and Healthcare, Geneva, CH

## Abstract

**Background:**

Forty years after Alma Ata, there is renewed commitment to strengthen primary health care as a foundation for achieving universal health coverage, but there is limited consensus on how to build strong primary health care systems to achieve these goals.

**Methods:**

We convened a diverse group of global stakeholders for a high-level dialogue on how to create an enabling ecosystem for disruptive primary care innovation. We focused our discussion on four themes: workforce innovation and strengthening; impactful use of data and technology; private sector engagement; and innovative financing mechanisms.

**Findings:**

Here, we present a summary of our convening’s proceedings, with specific recommendations for strengthening primary health care systems within each of these four domains.

**Conclusions:**

In the wake of the Astana Declaration, there is global consensus that high-quality primary health care must be the foundation for universal health coverage. Significant disruptive innovation will be required to realize this goal. We offer our recommendations to the global community to catalyze further discourse and inform policy-making and program development on the path to Health for All by 2030.

## Introduction

Forty years after Alma Ata, the global community has made enormous strides in poverty reduction and population health. However, we are still far from achieving *Health for All* [1,2]. Low- and middle-income countries (LMICs) continue to rely on health systems designed primarily for acute, episodic care, which are poorly equipped to meet the needs of communities across their lifetimes, including the growing burden of non-communicable diseases. There are universal challenges of healthcare access, quality, patient-centeredness and cost as well as persistent, unacceptable health inequities [2].

Our communities’ renewed commitment to primary health care (PHC) as the foundation for universal health coverage (UHC), ratified by the Astana Declaration [1], presents an imperative to reflect on past failings, current challenges, and future opportunities. Macro-economic strategies favoring privatization of public services along with loss of vision and political commitment to Alma Ata during the 1980s were associated with a retreat to selective primary health care and disease-specific vertical strategies as the core approach to improving health for LMICs. Consequently, an implementation gap between ambitious vision and operationalization widened in the ensuing decades [3]. There have been some promising exemplars during the time period from Alma Alta to Astana, including Cuba’s community-oriented primary health care system [4,5], Brazil’s family medicine programs [6], and large-scale community health worker programs such as Pakistan’s Lady Health Workers [7] and India’s Anganwadi workers [8]. With that being said, while the last half-century has seen immense growth in biomedical discovery for preventing, diagnosing and treating disease, there has been comparatively little innovation in our systems of health care delivery to spread these advances to improve access and quality of care and more equitable health outcomes.

To catalyze new thinking and innovation at this critical moment, the Harvard Medical School Program in Global Primary Care and Social Change, in partnership with Results for Development and the World Economic Forum, convened a group of diverse stakeholders for a high-level dialogue entitled *Primary Care 2030*. We aimed to identify priorities and forge new alliances across diverse stakeholders in order to foster an enabling ecosystem for disruptive innovations in primary care. Here, we summarize our convening, adding to the global discourse of how to achieve UHC by 2030. Full proceedings are accessible at https://primarycare.hms.harvard.edu/sites/g/files/mcu856/files/assets/Proceedings.pdf.

## An enabling ecosystem for primary care innovation

In the natural sciences, an ecosystem is a network of organisms with interdependent relationships between each other and the environment. Extrapolated to health care, a health ecosystem consists of a diverse group of stakeholders, including governments, private sector entities, civil society, health professionals, patients, and community members, working together to improve health. For our convening, we brought together a group of global stakeholders to catalyze innovative thinking across these interdependent relationships. We focused our discussion on four themes: workforce innovation and strengthening; impactful use of data and technology; private sector engagement; and innovative financing mechanisms.

### Workforce innovation and strengthening

LMIC health workforces struggle with shortages, inappropriate design, and insufficient support to meet the needs of the communities they serve [9]. Simply adding more health workers will not suffice; the workforce, from development to deployment, needs to be re-designed and professionalized, supported by strong and effective supervision, and appropriately compensated. As above, some historical examples of compelling programs from Cuba, Brazil, Pakistan, India, and other countries provide insights for future improvement and innovation.

During our convening, we agreed that team-based approaches, inclusive of cadres such as mid-level practitioners [10] and community health workers [2,11] with patients and their families actively engaged in healthcare delivery, will play a central role in scaling quality, patient-centered primary care services. Allied health professionals, such as health coaches, social workers, and patient navigators are also critical to the effectiveness of multidisciplinary teams [12]. Workforce strategies dependent upon physicians, or strategies that fail to create and enforce accountability, will continue to struggle to address the myriad access and quality challenges, especially on the front lines of care delivery.

### Data and technology for quality and scale

Despite present challenges, society’s digital transformation is a key opportunity for paradigmatic shifts in the scalability, safety, and quality necessary for UHC by 2030. Many LMICs still struggle with fundamental data capacity such as civil registration and vital statistics, significantly limiting their ability to provide quality primary care across patients’ lifetimes [13]. Transitioning from analog, paper registry systems to secure, interoperable digital records will enable longitudinal access to patients’ data across facility and community settings.

Coupling digital records with mobile applications used by patients and providers can augment data availability in real-time, enabling proactive care partnerships between patients and provider teams [14]. The adoption of user-centric technologies, including automated algorithmic clinical decision support and tools for real-time communication between patients and care teams, can help to scale quality primary care delivery, even in areas struggling with workforce shortages and other access barriers.

Several policy priorities emerged from our discussions of how to foster technology-enabled, team-based approaches to primary care. Firstly, there is a critical need for user-centered, open-source technology innovations with defined standards for data structure, security, and regulations that ensure interoperability, while simultaneously preserving patients’ rights to privacy and data ownership. Secondly, stakeholders must align around universal metrics for measuring the performance of primary care systems, such as those developed by the Primary Healthcare Performance Initiative (https://improvingphc.org/). Thirdly, we must standardize methods for evaluating innovations in primary care service delivery, ensuring that such evaluations receive funding priority on par with biomedical research. Finally, we must take advantage of the opportunities that real-time data availability offers, developing stronger data feedback loops and enabling rapid learning and improvement at the frontlines of care. This includes improvements in health workforce management, surveillance of disease burdens, and predictive planning for supply chains, among other opportunities.

### Meaningful engagement with private sector entities

Historically, much of the global discourse has championed public sector health service delivery in LMICs so as to build equitable health systems that can reach the most vulnerable. It is increasingly clear that UHC will require a more strategic engagement with the private sector [15]. Our conversations focused on how to cultivate trusting, impactful relationships between governments, non-government organizations, multilateral organizations, academia, and private businesses, to optimize equitable care delivery for patients and communities.

We agreed that the private sector must have a role in both innovation and direct service delivery. Governments historically struggle to catalyze research and development, while the private sector has bandwidth and resources to test and identify innovative best practices, including new service delivery processes, technologies, diagnostics, and medications. Working together, governments can collaborate with private sector actors to co-create products and services, accelerating their deployment and adoption at scale. To achieve this, governments need to develop national plans that provide consistent, predictable data regarding country demand, such that private sector entities can plan investments and contributions accordingly.

As service providers, private entities deliver health care services in areas throughout LMICs, including in remote, rural areas, where public sector services are limited in access and quality [16]. By working formally through public-private partnership models, governments and private entities can harness the benefits of both private and public sector service delivery. In doing so, governments can maintain strategic focus on core competencies of regulating and performance monitoring to ensure quality and equitable access.

### Innovative financing mechanisms for universal health coverage

We were particularly concerned with the financing and purchasing mechanisms of the public sector, which have historically posed significant barriers to innovation and quality service delivery, especially within the most impoverished and marginalized populations. We agreed that a major shift in financing from fragmented disease-specific mechanisms [3] to a more federated, equitable and efficient financing based on prepayment, pooling, and strategic purchasing, is a critical step for all countries on the path to UHC [17]. Working closely with the private sector, purchasing mechanisms within countries should be strengthened with strategic frameworks, using technology for predictive analysis of supplies, minimizing stock-outs, and maximizing access. Furthermore, transitioning toward health financing schemes that ensure financial protection and reward preventative care will be necessary to incentivize value-based integrated service delivery for all populations. This may require new capacities and legislation to enable strategic and value-based purchasing.

Furthermore, it is important to recognize that many governments have not historically had the autonomy or agency to determine their own financing and purchasing mechanisms, which have often been driven by the agendas of multilateral organizations, development banks, and non-governmental organizations. Accordingly, our group emphasized the importance of these international entities aligning amongst themselves with greater clarity on how development assistance for health can contribute more effectively to greater mobilization and better use of domestic financing, meeting the needs of local populations [18].

### Limitations

We brought together a diverse group of global stakeholders for a high-level dialogue on how to create an enabling ecosystem for disruptive primary care innovation. However, this was by no means representative of all stakeholders as key groups including patient representative groups, diversity in geographical distribution, and global south academic institutions were not present. Additionally, the *“private sector”* in our convening predominately represents multinational corporations (MNC), rather than private sector service delivery providers, which exist in many LMICs. Given the limited scope of the private sector representation in our convening, we did not discuss the “private sector-ization” of health care services; rather, we focused on how public-private partnerships in the form of how MNCs can meaningfully contribute to the national or sub-national PHC agenda, aligning objectives, strategies and shared indicators for success. The summary from our convening is not intended to be representative of all global voices, but rather, offer a concise perspective from some engaged voices.

## Conclusion

Much has changed in global health since Alma Ata in 1978. While we celebrate advances, we must acknowledge our shortfalls and the challenges ahead as we strive for UHC by 2030. Today, the global burden of disease is changing, health inequities are glaring, and past strategies will be insufficient for overcoming the significant barriers in our path. What has not changed, however, is the clarion vision of PHC as the basis for quality health service delivery, and its critical role in achieving UHC.

In this historic moment, we have summarized the insights of our convening, to catalyze further discourse. We firmly believe that UHC by 2030 is achievable, and that strong, technology-enabled, team-based PHC will be the foundation. It is time for a new visionary strategy that appropriately leverages past lessons, coupled with current opportunities, to advance primary care and finally achieve *Health for All*.
